# Gene Expression and Physiological Changes of Different Populations of the Long-Lived Bivalve *Arctica islandica* under Low Oxygen Conditions

**DOI:** 10.1371/journal.pone.0044621

**Published:** 2012-09-19

**Authors:** Eva E. R. Philipp, Wiebke Wessels, Heike Gruber, Julia Strahl, Anika E. Wagner, Insa M. A. Ernst, Gerald Rimbach, Lars Kraemer, Stefan Schreiber, Doris Abele, Philip Rosenstiel

**Affiliations:** 1 Cellbiology, Institute of Clinical Molecular Biology, Christian-Albrechts University Kiel, Kiel, Germany; 2 Functional Ecology, Alfred-Wegener-Institute for Polar and Marine Research, Bremerhaven, Germany; 3 Institute of Human Nutrition and Foodscience, Christian-Albrechts University Kiel, Kiel, Germany; Universidade de Brasília, Brazil

## Abstract

The bivalve *Arctica islandica* is extremely long lived (>400 years) and can tolerate long periods of hypoxia and anoxia. European populations differ in maximum life spans (MLSP) from 40 years in the Baltic to >400 years around Iceland. Characteristic behavior of *A. islandica* involves phases of metabolic rate depression (MRD) during which the animals burry into the sediment for several days. During these phases the shell water oxygen concentrations reaches hypoxic to anoxic levels, which possibly support the long life span of some populations. We investigated gene regulation in *A. islandica* from a long-lived (MLSP 150 years) German Bight population and the short-lived Baltic Sea population, experimentally exposed to different oxygen levels. A new *A. islandica* transcriptome enabled the identification of genes important during hypoxia/anoxia events and, more generally, gene mining for putative stress response and (anti-) aging genes. Expression changes of a) antioxidant defense: Catalase, Glutathione peroxidase, manganese and copper-zinc Superoxide dismutase; b) oxygen sensing and general stress response: Hypoxia inducible factor alpha, Prolyl hydroxylase and Heat-shock protein 70; and c) anaerobic capacity: Malate dehydrogenase and Octopine dehydrogenase, related transcripts were investigated. Exposed to low oxygen, German Bight individuals suppressed transcription of all investigated genes, whereas Baltic Sea bivalves enhanced gene transcription under anoxic incubation (0 kPa) and, further, decreased these transcription levels again during 6 h of re-oxygenation. Hypoxic and anoxic exposure and subsequent re-oxygenation in Baltic Sea animals did not lead to increased protein oxidation or induction of apoptosis, emphasizing considerable hypoxia/re-oxygenation tolerance in this species. The data suggest that the energy saving effect of MRD may not be an attribute of Baltic Sea *A. islandica* chronically exposed to high environmental variability (oxygenation, temperature, salinity). Contrary, higher physiological flexibility and stress hardening may predispose these animals to perform a pronounced stress response at the expense of life span.

## Introduction

The Ocean Quahog, *Arctica islandica*, is regarded a prodigious new study object in aging research. It is the longest-lived non colonial animal species on earth with an extreme tolerance to low environmental oxygen levels. Individuals were found to live >400 years [1] and to survive extended periods without oxygen (50% survival rate after 55 days of anoxia; Theede et al. [2]). The species' pronounced ability to tolerate low oxygen concentrations may be part of the underlying mechanisms to slow physiological ageing. *A. islandica* is an oxyconforming organism whose metabolic rate is attuned to the environmental oxygen level and thus slows when oxygen is scarce and accelerates when it is abundant [3]. Previous studies in the laboratory showed that during periods of low oxygen, the animals enter a metabolically depressed state (MRD) down to 10%, and under longer anoxic conditions even down to 1% of their normoxic metabolic activity [4]. This also means abatement of reactive oxygen species (ROS) formation in quahog tissues at low oxygen levels, as lately shown for isolated gill tissues [5]. *A. islandica* can self-induce MRD by spontaneously burrowing into the sediment, closing the shell and exposing their soft tissues to hypoxic and even completely anoxic conditions for 1–7 days [5], [6]. In the Iceland population, the duration and frequency of MRD phases as well as burrowing depth varies between seasons, with colder winter temperature causing increasing burrowing frequency [5].

For several animals, including turtles, bats, the naked mole rat or vertebrate hibernators, life prolongation as a side effect of anoxia/hypoxia- and general stress tolerance, as well as metabolic down regulation has been reported [7], [8]. Physiologically this behavior might slow senescence in several ways. Firstly, it involves a slowdown of metabolic reactive oxygen species (ROS) production in the depressed state [5], [9] whereby it would decelerate the age-related accumulation of ROS induced oxidative cellular damage. Normally, generation of ROS accelerates as normoxic respiration is resumed following the shut-down phase [10]. This may, however, not always be the case. In *A. islandica* for example oxidative burst during re-oxygenation does not seem to occur, as shown for isolated gill tissue of Icelandic individuals [5]. Further, in several ectotherms, frequent hypoxia/re-oxygenation cycles were found to trigger antioxidant protection [11]. Snails, snakes and turtles increase antioxidant capacity and heat-shock protein concentration during periods of anoxia, which is thought to prepare the animal for the oxidative burst during re-oxygenation [7], [11], [12]. Abele [13] proposed such an anticipatory and preparative stress response to also occur in burrowed *A. islandica* and suggests that antioxidant genes might be induced during MRD episodes. However, so far no changes of the antioxidant capacities (antioxidant enzyme activities and glutathione) were observed in *A. islandica* from the longer lived German Bight or Icelandic populations during self-induced phases of MRD or in animals incubated under experimental hypoxia or anoxia and during MRD [5], [14].

While a maximum age of 410 years is reported for the Iceland population [1], *A. islandica* from the Baltic Sea population do not even reach 50 years of age, as determined by shell ring counts [15]. Due to the enclosed character and the limited water exchange through inflow from the North Sea through the Kattegat into the marginal Baltic Sea, these *A. islandica* populations are exposed to frequent and prolonged environmental hypoxia and anoxia episodes. In contrast environmental conditions are more constant around Iceland and the German Bight with low annual fluctuations in oxygen content or salinity [15]. Oxygen minimum zones in the Baltic Sea appear especially during summer when water stratification stabilizes and oxygen is rapidly depleted by bacterial degradation of organic matter below the pycnocline (10–15 m) [16]. *A. islandica* of the Baltic Sea is known to be extremely anoxia tolerant. In late summer 1981 an extraordinary and wide ranging oxygen depletion killed most (90%) of the benthic fauna below the halocline (>20 m) in Kiel Bay. The few survivors were the low oxygen tolerant species *Arctica*, *Astarte, Corbula, Halicryptus* [17]. Theede et al. [2] investigated the anoxia tolerance of Baltic Sea *A. islandica* experimentally and reported the highest survival rate of all investigated species with an Lt_50_ ( = 50% survival) after 55 days of anoxia exposure. While theoretically, as described above, increased anoxia/hypoxia tolerance could lead to increased longevity, this does not seem to be the case in Baltic Sea *A. islandica*. To the contrary, frequent self induced and environmental conditions of low oxygen may have caused stress hardening as a physiological response in Baltic Sea *A. islandica*. Especially in stressful environments, i.e. with high fluctuations in several environmental parameters, shorter periods of subcritical, tolerable stress can induce up-regulation of stress response genes, often, at the costs of growth rate, cellular maintenance and life expectancy (for review see [18]).

The present paper aims to understand whether the different populations of *A. islandica* with longer and shorter maximum life spans (MLSP) show different responses when facing hypoxia/anoxia and elucidate their genomic response during MRD and re-oxygenation. To this end, RNAseq data of *Arctica islandica* were generated to increase the amount of *A. islandica* sequence information in the public databases and to have a number of genes at hand for the investigation of molecular hypoxia/anoxia responses of individuals from the longer lived German Bight population (MLSP ∼150years) compared to specimens from the shorter lived Baltic Sea population (MLSP ∼40years). The investigation of molecular changes was accompanied by the analysis of cellular antioxidant capacities on the protein level as well as protein damage and apoptotic activity as markers for oxidative stress. In respect to the above described changes in ROS production and metabolic rate during anoxia/hypoxia and re-oxygenation in *Arctica islandica* and other mollusks, we especially focused on genes important for oxygen sensing, antioxidant defense, stress response and anaerobic metabolism as we anticipated changes most likely to occur within such pathways. We expected faster pO_2_ related responses on the gene expression and antioxidant activity/protein level in Baltic Sea *A. islandica* as part of a possible adaptation to a highly fluctuating environment, which on the one hand will abate oxidative stress related cellular damage but on the other hand may mean increased costs to maintain cellular homeostasis compared to animals of the German Bight population, presumably on the expense of life span.

## Methods

### Animal sampling and experiments for transcriptome generation

Seven independent transcriptomal data sets of *A. islandica* were generated using 454 technology (454 Life Sciences, Branford, CT, USA). To maximize the diversity of expressed stress- and immune-related transcripts in the experimental material, *A. islandica* individuals were exposed to different stressors and two tissues (gill, digestive gland) were used for RNA extraction. Briefly, *A. islandica* individuals were collected in March 2008 in the Baltic Sea (Germany) at the sampling station “Süderfahrt” (54°32.6′N, 10°42.1′O) in 20 m water depth with a hydraulic dredge. Animals were kept in a constant temperature room at 10°C with a flow-through of natural unfiltered seawater from Kiel Bay. After 1 week of acclimation, animals were exposed to different experimental conditions (2 animals each): high temperature (20°C); anoxia: wrapped in a double layer of parafilm, and injury: cracking the shell. Control animals were kept at 10°C in flow-through seawater. After 5 days, the animals were taken from each experiment, dissected and tissues frozen in liquid nitrogen. From these experiments digestive gland and gill tissue was used for sequence generation. To further enhance the number of transcripts, gill tissue of three young (27, 19 and 22 years of age) and two old (148 and 150 years of age) *Arctica islandica* individuals from an Icelandic population were used for transcriptome generation, which were collected by J. Strahl in July 2004 and May 2005 Northeast of Iceland (66°01.54′N, 14°50.98′W) between 14 and 22 m water depth using a hydraulic dredge. Animal processing and age determination by year ring counts in the shell is described in detail in Strahl et al. [19]. Several studies verified the growth layers in the shell of *A. islandica* as annual rings by means of oxygen and carbon isotopes [20], [21]. No specific permits were required for the described sampling as the locations are not protected and sampling did not involve any endangered or protected species.

The samples were ground in liquid nitrogen and total RNA extracted using the Qiagen RNeasy kit in combination with the QiaShredder from Qiagen (Hilden, Germany) according to manufacturer's instructions. The mRNA was purified using the Oligotex mRNA purification kit (Qiagen, Hilden, Germany) and cDNA generated with the SMART cDNA synthesis kit (Clontech Laboratories, Palo Alto, CA, USA). Quality control in each extraction step was investigated using gel electrophoresis and a nanodrop spectrophotometer (Peqlab, Erlangen). cDNA was further checked with a Bioanalyzer 2100 (Agilent Technologies, USA).

### 454 sequencing, EST assembly

Samples were prepared for 454 sequencing according to the manufacturers protocol and sequenced on a Genome Sequencer FLX system (454 Life Sciences, Branford, CT, USA) with FLX and Titanium chemistry. The resulting 454 sequences were extracted from FLX output files using the ‘sffinfo’ script from Roche (See Table S1). Subsequently sequences were again quality controlled and cleaned for primer and adapter sequences (Smart primer sequences and 454 adapters), polyA tails as well as bases on sequence ends with low quality scores (≤10) by ‘seqclean’, ‘cln2qual’ (TGI - The Gene Index Project) before the assembly. Reads smaller than 50 bp after the quality control and cleaning were excluded from further sequence assembly and annotation (Table S1). The trimmed reads were assembled using the Celera assembler (Version 5.3) with custom settings, as a first step. Afterwards the resulting contigs and singletons were further assembled in multiple rounds using the TGICL (Cap3) assembler. The ‘minimum overlap length’ varied between 40 and 300 bp and the ‘minimum overlap identity’ between 85 and 100%. At last all initial reads were mapped against the generated contigs using AMOScmp (http://sourceforge.net/apps/mediawiki/amos/index.php?title=AMOS), which resulted in the final contigs.

### Contig annotation

Putative gene names and protein domains were assigned to the assembled contigs of the *A. islandica* transcriptome using the BLASTx algorithm against the UniprotKB/Swissprot and UniRef100 database of the UniProt Knowledgebase (UniProtKB, http://www.expasy.org/sprot/) with a cut off e-value of e ≤10^−3^, as well as tBLASTx (e≤10^−3^) against the NCBI nt database (http://www.ncbi.nlm.nih.gov). To identify conserved domains, the assembled contigs were run via InterProScan [22]. Gene Ontology (GO) terms were deduced from the BLAST and InterProScan results and sorted into the immediate subcategories for ‘molecular function’, ‘cellular component’ and ‘biological process’. Sequences and associated information of read cover and annotation were loaded into the transcriptome software tool T-ACE [23] for further analysis.

### Identification of candidate genes

Orthologs of genes related to anoxia/hypoxia tolerance, stress response, the ageing process and the immune system were searched via key words and protein domain structure in the *A. islandica* database in T-ACE. Identified transcripts were visually inspected and re-blasted by hand via the NCBI nt (tBLASTx) and UniprotKB/Swissprot (BLASTx) database. Protein domains were re-evaluated using SMART [24] and transmembrane domains specifically analyzed with the TMHMM Server v. 2.0 (www.cbs.dtu.dk/services/TMHMM-2.0/). To confirm correct assembly, primers targeting selected candidate genes for hypoxia/anoxia tolerance and stress response were designed using Primer3 [25] and PCR was performed with Advantage Taq 2 polymerase (Clontech, Germany) and the appropriate PCR conditions (Table S2). Briefly we selected contigs with putative function for antioxidant defense: Catalase (Cat), Glutathione peroxidase (GPx), manganese Superoxide dismutase (Mn-SOD) and copper-zinc Superoxide dismutase (Cu/Zn SOD); oxygen sensing and general stress response: Hypoxia inducible factor alpha (HIF-α), Prolyl hydroxylase (PHD) and Heat-shock protein 70 (HSP70); as well as anaerobic capacity: Malate dehydrogenase (MDH), Octopine dehydrogenase (ODH). PCR products were evaluated by gel electrophoresis to confirm the presence and size of the transcripts and after extraction and purification from the gel sequenced using the BigDye™ Terminator v1.1 Cycle Sequencing Kit (Applied Biosystems, Life Technologies, USA). Resulting sequences were assembled using Sequencher version 4.5 (GeneCodes, USA).

### Expression analysis of selected *A. islandica* genes in Baltic Sea and German Bight individuals under environmentally forced and self-induced hypoxia/anoxia and re-oxygenation

**Figure 1:**
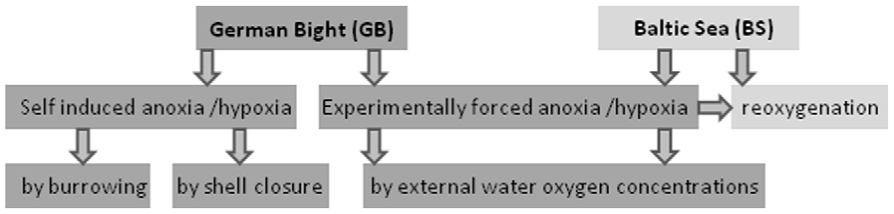
Flow diagram of the experimental design to generate the gill samples of *A. islandica* investigated in the present study from animals under self induced and experimentally forced hypoxia and anoxia as well as after re-oxygenation.

Expression levels of the selected candidate genes were investigated in gill samples of *A. islandica* individuals from the Baltic Sea and German Bight population exposed to different oxygen conditions (Fig. 1) using quantitative real time PCR (qRT-PCR). Baltic Sea *A. islandica* individuals were collected at the sampling station “Süderfahrt” in February 2011. Animals were transported to the Geomar Helmholtz Centre for Ocean Research (Kiel, Germany) and kept for 3 weeks in a 60 L tank with 6°C flow-through seawater from Kiel Bay to recover from the sampling. Animals were fed twice a week with *Rhodomonas spec*.. Feeding was stopped 2 days prior the incubation experiment to avoid contamination of the experimental aquaria. For the incubation under different oxygen concentrations, animals were transferred to 2 L plastic aquaria (21 aquaria with 4 animals each) and acclimated for 8 h to the experimental conditions. Using different gas mixtures, animals were then exposed to normoxia (21 kPa), hypoxia (2 kPa) or anoxia (0 kPa) by constantly bubbling the water with either air, a pre-mixed gas (2% O_2_:98% N_2_, Air Liquide, Germany), or N_2_, respectively. Oxygen concentrations in the hypoxia and anoxia treatment were monitored throughout the experiment in one reference aquaria per treatment using non-invasive oxygen sensor spots (PreSens, Germany). After 3.5 days one animal per aquaria was dissected and gill tissue frozen in liquid nitrogen until further analysis. The hypoxic and anoxic aquaria were then bubbled with air to re-establish normoxic conditions, which were attained after 20 min. 1 and 6 h after normoxia was retained, samples for re-oxygenation responses were taken as described above.

We were also interested to analyze population specific differences in gene expression response under hypoxia/anoxia as well as between experimentally forced and self-induced hypoxia/anoxia. Therefore gill samples from previous experiments with animals from a German Bight population were included in the study. Animal collection and experiments are described in detail in Strahl et al [5], [14]. As experimentally forced samples, gill tissues from German Bight individuals which were incubated in the same manner and oxygen concentrations as described above for the Baltic Sea population were used. For animals under self-induced hypoxia/anoxia, gill tissues of animals after 24 h anoxia induced by shell closure were investigated. In these animals oxygen concentration was monitored constantly in the mantle cavity water by oxygen optodes (Presens, Germany; [5]; [26]). As control animals, individuals were used which showed fluctuating pO_2_ concentration over the same time span [5]. Further gill tissue of individuals, which were burrowed in the sediment for 3.5 days was investigated together with normoxic control individuals that were constantly visible at the surface (non-burrowed).

Total RNA of the different gill samples was extracted as described above. cDNA was generated using the RevertAid Premium First Strand cDNA Synthesis Kit (Fermentas, Germany) according to manufacturer's instructions. Gene expression was investigated using SYBR Green (Power SYBR® Green PCR Master Mix, Applied Biosystems, USA) on a 7900HT Fast Real-Time PCR System (Applied Biosystems, USA). In total 45 PCR cycles with an elongation time of 60 sec and an annealing temperature of 60°C were performed. PCR cycling conditions were 50°C for 2 min, 95°C for 10 min, followed by 40 cycles of 95°C for 15 s, 60°C for 1 min. Samples were run in triplicates. Primers were designed using Primer3 [25] (Table 1) and for each primer a melting curve of the PCR product was performed to ensure the absence of artifacts. Primer efficiency was assessed by serial template dilution. The comparative CT method (delta Ct) for the relative quantification of gene expression was used. To find a suitable reference gene for normalization we applied Normfinder (http://www.mdl.dk/publicationsnormfinder.htm). As input data the logarithmic CT values of the different genes and groups transformed into linear values with the respective efficiency E (Table 1), calculated by E = 10∧(−1/slope of the dilution curve), was used. Normfinder suggested 18S rRNA, 28 s rRNA and HSP 90 as most suitable reference genes and a geometric mean of the three genes was used for gene normalization.

**Table 1:** Forward and reverse primer sequences used for quantitative real time PCR analyses.

Category	Accession	Candidate gene	5′-3′ forward primer	5′-3′ reverse primer	Amplicon length (bp)	Primer efficiency
Antioxidant system	HE792873	Catalase (cat)	TTCCCACCAAATCCCAGT	ACAAAAATGCCTCCAAGACG	333	2.25
	HE792874	Glutathione peroxidase (GPX)	GGGCTCATCATTTTGTGTGG	CTCAGTAGGTCGCCGCATT	256	1.66
	HE792875	Superoxide dismutase (Cu/Zn SOD)	GCCGTATTTATCGTGGAAGC	GTCAGCGTGGTCATCGTG	297	1.87
	HE792877	Superoxide dismutase (Mn SOD)	GCCAAATAACGGAACTAAACC	AATGCCACTGAGGAAAAGC	375	2.02
Anaerobic metabolism	HE792878	Octopine dehydrogenase (ODH)	TCACTCTCTCGCCGTTGTTA	GTCCATCTTTCAGCCTCGTC	247	2.34
	HE792879	Malate dehydrogenase (MDH)	CTCCATACCCTCCCTTCTCG	TCGGAAATGACCAGCAAATC	203	1.99
Oxygen sensing	HE792881	Hypoxia inducible factor alpha (HIF alpha)	CCATCCAAACATAACCACCA	CAATCCCTCCCACACTTCTT	310	2.18
	HE792884	HIF prolyl hydroxylase (PHD)	CGACTTAGCGATTTCCAGGT	TTGGCATCTTCACATTTCTTG	276	1.96
Stress response	HE792890	HSP90	GGGCTTGTCTTCCTCCTTTT	GGTCAGTTTGGTGTGGGTTT	375	1.94
	HE792888	HSP70	GGTGAATGTCTGGGTCTGCT	TTGTGCTGGTCGGTGGTT	287	1.93
Housekeeper	HE962431	18 S	TGGTGCTCTTGACTGAGTGTCTCG	GGCAAATGCTTTCGCTGTAGTTC	247	1.95

### Assays of antioxidative defense, oxidative damage and apoptosis

To investigate the occurrence of oxidative stress in phases of metabolic rate depression in the two populations and under the different oxygen regimes on the cellular level, the activity of catalase, glutathione peroxidase and the concentration of total glutathione were measured in gill tissue of the respective specimens. For gill homogenates, about 70 mg tissue was homogenized on ice in 140 µl ice-cold phosphate buffered saline (PBS; ratio 1∶3) and centrifuged at 20800× g at 4°C for 10 min. The supernatant was transferred into a fresh tube and stored at −80°C until further analysis. Total protein was determined with the bicinchoninic acid (BCA) assay (Pierce, USA) according to manufacturer's instructions.

### Catalase Measurement

The activity of the antioxidant enzyme catalase was analyzed according to the method described by Johansson and Borg [27]. Due to its peroxidative function catalase transforms methanol into formaldehyde depending on an optimal concentration of hydrogen peroxide. Formaldehyde production was measured by using 4-amino-3-hydrazino-5- 1,2,4 – triazole (Purpald) as chromogen. For quantification a standard curve of formaldehyde was applied where 1 unit of catalase was defined as the amount of enzyme producing 1 nmol formaldehyde per min at 25°C. The catalase activity of each sample was related to the corresponding protein content. Internal standards were included in each measurement.

### Glutathione Measurement

The total glutathione (tGSH) measurement was performed according to the method published by Vandeputte et al. [28]. Initially GSSG is reduced by glutathione reductase to GSH which in turn reacts with DTNB to generate 2-nitro-thiobenzoate (TNB), a chromogenic thiol compound that can be detected at 415 nm in a plate reader (iEMS, Labsystems, Finland). For quantification a standard curve of GSH (0.5–80 µmol/l) was used. The glutathione content of each sample was related to the corresponding protein content. Internal standards were included in each measurement.

### Glutathione Peroxidase Measurement

Glutathione Peroxidase (GPx) activity was determined using a commercially available test kit (Enzo Life Sciences, Loerrach, Germany). The measurement was performed according to manufacturers' instructions. GPx reduces hydrogen peroxide to water by oxidizing GSH to GSSG. GSSG in turn is reduced to GSH by glutathione reductase and simultaneous oxidation of NADPH+H^+^ to NADP^+^. The oxidation of NADPH+H^+^ to NADP^+^ results in a decrease of the absorbance at 340 nm which is directly proportional to the GPx activity. The GPx activity of each sample was related to the corresponding protein content. Internal standards were included in each measurement.

### Determination of protein carbonyls

Oxidation of proteins can lead to carbonyl derivates and the occurrence of protein carbonyls in cells and tissues is a widely accepted marker macromolecular oxidative damage and the occurrence of oxidative stress. For the investigation of oxidative cell damage during hypoxia, anoxia and re-oxygenation, the protein carbonyl content was assessed in gill tissue of Baltic Sea individuals using the Oxiselect™ Protein Carbonyl ELISA Kit (Cell Biolabs, USA). Gill tissue was homogenized in ice-cold PBS with a glass homogenizer (Gerresheimer, USA). Samples were centrifuged for 10 min and 10000 g at 4°C and the supernatant transferred into a new reaction vial. Protein concentration was determined by the DC protein assay (Bio-Rad, Germany) and samples diluted to a concentration of 10 µg/mL. Protein carbonyls were determined according to the manufacturer's guidelines on a GENios Pro Microplate Reader (Tecan, Switzerland) at 492 nm.

### Apoptotic cell death

To investigate whether changing oxygen concentrations trigger apoptosis, caspase activity was measured in Baltic Sea individuals after 3.5 days hypoxia or anoxia, followed by 1 h and 6 h re-oxygenation, using the Caspase-Glow 3/7 Assay kit (Promega, USA). Gill tissue was homogenized in lyses buffer (25 mM HEPES, 5 mM MgCl_2_, 1 mM EGTA and 1 tablet/10 mL complete protease inhibitor cocktail (Roche, Germany)). Caspase 3 activity, as a marker of apoptosis, was measured as previously described by Strahl et al [29]. Luminescent intensity was determined on a GENios Pro Microplate Reader (Tecan, Switzerland) and normalized to the sample protein concentration, determined with the DC protein assay (Bio-Rad, Germany). Data are displayed as relative fluorescent units (RLU)/mg protein.

### Statistics

Statistical analysis was undertaken using GraphPad Prism (Version 5). All data sets were tested for normality (Kolmogorov-Smirnov) and homogeneity of variances (Bartlett) prior to analysis and if necessary LOG-transformed. One-factorial variance of analysis (non-parametric Kruskal-Wallis-Test for non-Gaussian distribution) with Bonferroni's Post-Hoc-Test (Dunn's test for non-Gaussian distribution) was used to analyze differences in gene expression, antioxidant enzyme activity, protein carbonyls and apoptotic activity between three or more experimental groups. Differences between two groups were tested using the t-test after Student (Mann-Whitney-Test for non-Gaussian distribution, Welch correction for non-equal variances).

## Results

### Transcriptome overview

In order to mine for a high repertoire of expressed genes, sequences from different populations, tissues and stress-challenges as well as distinctly old *A. islandica* individuals were generated on a Genome Sequencer FLX system (454 Life Sciences, USA). A total of 809327 reads with an average length of 295 bp passed the quality control and filtering and entered the assembly pipeline (Figure S1 and Table S1). From these, 710849 of the 809327 reads could be assembled into 35551 contigs with an average length of 626 bp (Figure S1) and an n50 value of 692 bp. 98478 reads could not be assembled and remained as singletons. The GC-content of the transcriptome was 36.92% and the AT/GC-ratio 1.70. The transcriptome project is deposited as SRA study ERP001285 and Bioproject 89475 at the European Nucleotide Archive (ENA). Assembled transcriptome sequences are deposited in the ENA as Transcriptome Shotgun Assembly (TSA) and accession numbers are given in the tables.

### Transcriptome annotation

To associate a putative function to the assembled contigs and search for candidate genes important for hypoxia/anoxia tolerance, stress response, involved in the aging process and immune functions, *A. islandica* contigs were blasted against public databases (UniprotKB/Swissprot and UniRef100, NCBI nt). 44% of contigs with sequence sizes >500 bp could be annotated either with BLAST matches, GO terms or proteins domains (Table 2). The subsequent key word and BLAST searches led to identification of a number of genes which may be important in hypoxia/anoxia tolerance as well as general stress response (Table 3, Table S3). Of these genes, contigs with putative function for Catalase (Cat), Glutathione peroxidase (GPx), manganese Superoxide dismutase (Mn-SOD), copper-zinc Superoxide dismutase (Cu/Zn SOD), Hypoxia inducible factor alpha (HIF-α), Prolyl hydroxylase (PHD), Heat-shock protein 70 (HSP70), Malate dehydrogenase (MDH) and Octopine dehydrogenase (ODH) were further investigated for expression changes.

**Table 2:** General characteristics of the *Arctica islandica* transcriptome contig annotation.

	all sequences	100–500 bp	>500 bp
total number of contigs	35551	16938	18276
contigs with BLAST matches	11676	4004	7662
assigned GO terms	6149	1975	4170
contigs with InterProScan matches	8636	2759	5873
contigs without matches	23164	12663	10174
% annotated	34.84	25.24	44.33

**Table 3:** BLAST annotation of candidate genes important for anoxia/hypoxia tolerance and general stress response identified in the *A. islandica* transcriptome.

		Contig information			Best blast hit (UniprotKB/SwissProt)				Best blast hit (nr/nt NCBI)			
*Category*	*Gene name*	*Accession*	*Contiq length (bp)*	*Nr. of reads in contig*	*Accession*	*Description*	*Organism*	*E-Value*	*Accession*	*Description*	*Organism*	E-value
Hypoxia/Anoxia	Catalase	HE792873*	2145	137	P00432	Catalase	*Bos taurus*	0.0	HM147935.1	Catalase (Cat-2)	*Crassostrea hongkongensis*	0.0
	Glutathione peroxidase (GPX)	HE792874*	1210	678	P21765	Epididymal secretory glutathione peroxidase	*Mus musculus*	2,00E-35	HQ437317.1	Selenium-dependent glutathione peroxidase	*Meretrix meretrix*	5,00E-133
	Superoxide dismutase (Cu/Zn SOD)	HE792875*	1934	381	Q54G70	SOD [Cu-Zn]	*Dictyostelium discoideum*	8,00E-11	XM_001651807.1	Superoxide dismutase	*Aedes aegypti*	3,00E-08
		HE792876	1270	179	P80566	Superoxide dismutase [Cu -Zn]	*Venerupis philippinarum*	6,00E-56	GQ384412.1	Cu/Zn-superoxide dismutase	*Venerupis philippinarum*	2,00E-65
	Superoxide dismutase (Mn SOD)	HE792877*	1682	86	P09671	SOD [Mn]	*Mus musculus*	5,00E-83	GQ202272.1	Manganese superoxide dismutase	*Laternula elliptica*	4,00E-105
	Octopine dehydrogenase (ODH)	HE792878*	461	2	Q8T882	Tauropine dehydrogenase	*Arabella iricolor*	2,00E-08	AB197036.1	Odh mRNA for octopine dehydrogenase	*Pseudocardium sachalinensis*	6,00E-40
	Malate dehydrogenase (MDH)	HE792879*	1497	616	Q5ZME2	MDH	*Gallus gallus*	1,00E-131	XM_002424763.1	Malate dehydrogenase putative	*Pediculus humanus corporis*	2,00E-150
		HE792880	1390	119	P40926	Malate dehydrogenase, mitochondrial	*Homo sapiens*	1,00E-130	AF218064.1	Malate dehydrogenase precursor	*Nucella lapillus*	6,00E-157
	Hypoxia inducible factor alpha (HIF alpha)	HE792881*	4322	96	Q61221-2	Isoform 2 of Hypoxia-inducible factor 1-alpha	*Mus musculus*	1,00E-96	HM441076.1	Hypoxia-inducible factor 1 alpha (HIF-1a)	*Crassostrea virginica*	9,00E-140
	Aryl hydrocarbon receptor nuclear translocator (HIF beta/ARNT)	HE792882	762	2	P27540-3	Aryl hydrocarbon receptor nuclear translocator	*Homo sapiens*	2,00E-37	FJ807919.1	Hypoxia inducible factor 1 beta	*Litopenaeus vannamei*	9,00E-50
	Von Hippel-Lindau (VHL)	HE792883	358	2	P40338	Von Hippel-Lindau disease tumor suppressor	*Mus musculus*	6,00E-04	XM_003441465.1	Von Hippel-Lindau disease tumor suppressor-like	*Oreochromis niloticus*	0.015
	HIF prolyl hydroxylase (PHD)	HE792884*	567	3	Q8BG58	Transmembrane prolyl 4-hydroxylase	*Mus musculus*	1,00E-31	XM_001185831.1	PH-4 protein	*Strongylocentrotus purpuratus*	3,00E-47
	Peroxiredoxin	HE792885	1256	103	P30044	Peroxiredoxin-5, mitochondrial	*Homo sapiens*	6,00E-40	EU734750.1	Peroxiredoxin V	*Laternula elliptica*	2,00E-50
		HE792886	1212	17	Q90384	Peroxiredoxin	*Cynops pyrrhogaster*	6,00E-82	HQ166838.1	Thioredoxin peroxidase	*Cristaria plicata*	5,00E-114
	Thioredoxin	HE792887	2366	61	O96952	Thioredoxin	*Geodia cydonium*	6,00E-28	Y17147.1	Thioredoxin	*Geodia cydonium*	4,00E-30
Stress response	HSP70 family^a^	HE792888*	2379	482	Q9U639	Heat shock 70 kDa protein cognate 4	*Manduca sexta*	0.0	HQ256748.1	Heat shock protein 70	*Meretrix meretrix*	0.0
		HE792889	4431	130	Q90593	78 kDa glucose-regulated protein	*Gallus gallus*	0.0	AB122065.1	78kDa glucose regulated protein	*Crassostrea gigas*	0.0
	HSP90 family^a^	HE792890*	2826	1603	O02705	Heat shock protein HSP 90-alpha	*Sus scrofa*	0.0	EU831278.1	Heat shock protein 90	*Laternula elliptica*	0.0
		HE792891	2967	29	P08113	Endoplasmin	*Mus musculus*	0.0	AB262084.1	Glucose-regulated protein 94	*Crassostrea gigas*	0.0
		HE792892	1017	154	Q7PT10	Heat shock protein 83	*Anopheles gambiae*	1,00E-107	GQ503177.1	Heat shock protein 90	*Phascolosoma esculenta*	2,00E-138

a For HSPs only contigs with >100reads were selected for display. Additional contigs with high similarity to HSPs are present in the transcriptome.

* Genes investigated for differential expression in *A. islandica* gill tissue.

Further, a high number of contigs, homologous to known genes ascribed to the ageing process, stress responses, immune functions, autophagy and apoptosis in other organisms were detected in the transcriptome (Table S4). We could identify contigs with high similarity to the ageing associated sirtuin genes and DNA repair protein MutL, components of the innate immune system including cellular receptors and transcription factors, as well as components of the autophagic and apoptotic machinery which may be important to retain homeostasis under stressful conditions.

### Gene expression analysis

**Figure 2:**
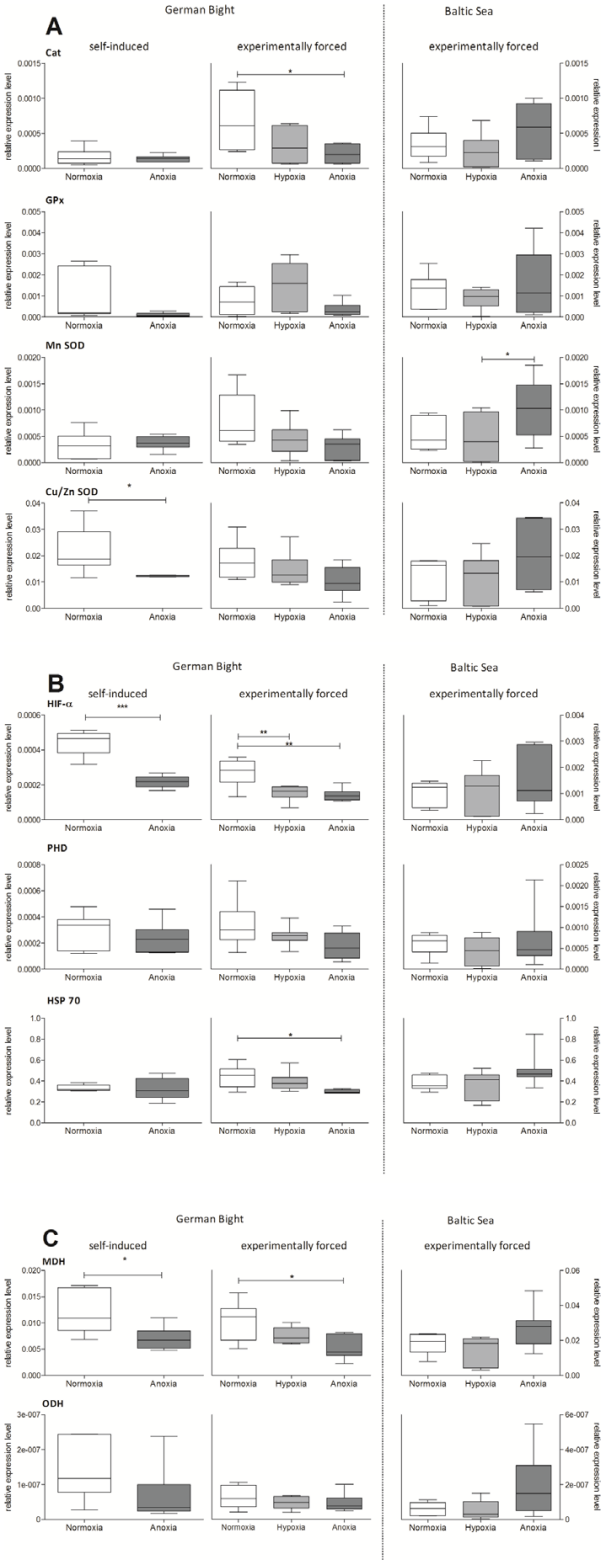
Quantitative expression of candidate genes for A) antioxidative defense: Catalase (Cat), Glutathione peroxidase (GPx), manganese Superoxide dismutase (Mn-SOD), copper-zinc Superoxide dismutase (Cu/Zn SOD); (B) oxygen sensing and stress response: Hypoxia inducible factor alpha (HIF-α), Prolyl hydroxylase (PHD), Heat-shock protein 70 (HSP70); C) anaerobic capacity: Malate dehydrogenase (MDH), Octopine dehydrogenase (ODH). Expression was assessed by q-RT PCR in gill tissue of Baltic Sea (BS) and German Bight (GB) *A. islandica* individuals exposed for 3.5 days to Normoxia, Hypoxia and Anoxia (experimentally forced) and German Bight individuals with 24 h self-induced normoxia and anoxia by shell closure (self-induced). Expression levels were normalized using the geometric mean of 18S rRNA, 28S rRNA and HSP 90, which were selected as most stable reference genes by Normfinder. Number of n per group = 6–8. Values are significantly different with *p<0.05, ** p<0.01 and *** p<0.001 (t-test for self-induced GB samples and one-way ANOVA for GB and BS experimentally forced data).

**Figure 3:**
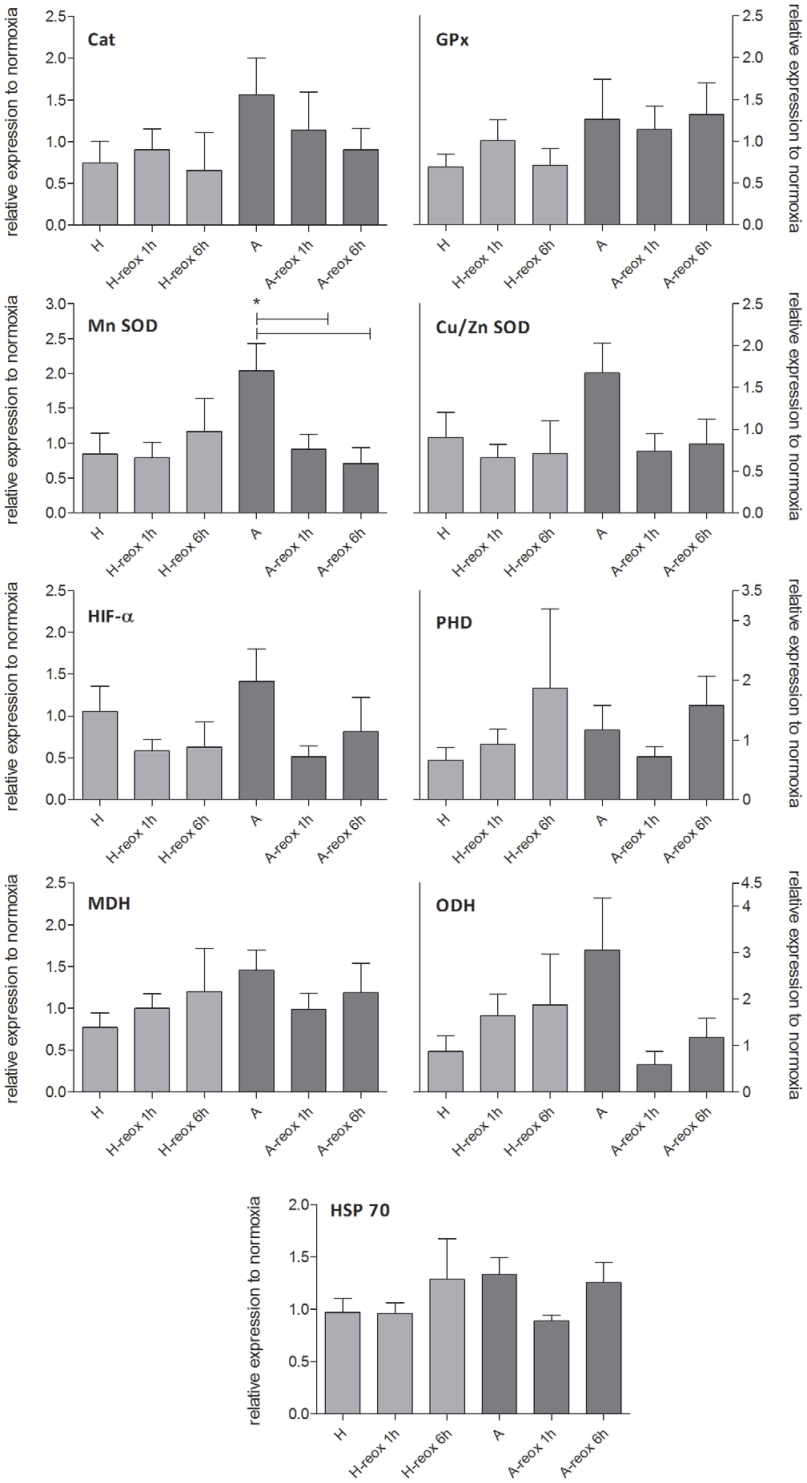
Quantitative expression of candidate genes for antioxidative defense (Catalase (Cat), Glutathione peroxidase (GPx), manganese Superoxide dismutase (Mn-SOD), copper-zinc Superoxide dismutase (Cu/Zn SOD)), oxygen sensing and stress response (Hypoxia inducible factor alpha (HIF-α), Prolyl hydroxylase (PHD), Heat-shock protein 70 (HSP70)) and anaerobic metabolism (Malate dehydrogenase (MDH), Octopine dehydrogenase (ODH)) of Baltic Sea *A. islandica* individuals exposed for 3.5 days to Normoxia, Hypoxia (H) and Anoxia (A) followed by 1 h (-reox 1 h)and 6 h (reox 6 h) of re-oxygenation. Expression was assessed by q-RT PCR and normalized (delta CT) using the geometric mean of 18S rRNA, 28S rRNA and HSP90, which were selected as most stable reference genes by Normfinder. Data are displayed as delta delta CT values relative to the respective normoxic control: Hypoxia and Anoxia to Normoxia; Hypoxia 1 h reox and Anoxia 1 h reox to Normoxia 1 h control; Hypoxia 6 h reox and Anoxia 6 h reox to Normoxia 6 h control. * = significantly different from each other (p<0.05, one-way ANOVA). Number of n per group = 6–8.

Gene expression changes of the selected candidate genes important for hypoxia/anoxia tolerance and general stress response were investigated in gill tissue of German Bight and Baltic Sea *A. islandica* exposed to different oxygen regimes, either experimentally forced or self-induced. Overall expression changes were quite variable and, in most cases, did not reach significance. In animals of the German Bight population, expression levels generally declined under oxygen reduced conditions (Fig. 2 A–C). Especially Cat, HIF-α, HSP70 and MDH were significantly down-regulated in 3.5 days anoxically incubated individuals compared to normoxic individuals. Contrary, in individuals of the Baltic Sea population the opposite response occurred. Expression levels of most of the investigated genes were higher in anoxically incubated than normoxically or hypoxically treated animals. This was especially pronounced for Cat, Cu/Zn SOD, HIF-α and MDH, but did only reach significance in MnSOD (Fig. 2 A–C). Re-oxygenation of Baltic Sea individuals after 3.5 days of anoxia led to a general return of the expression back to normoxic levels (Fig. 3). Such decrease was stronger for MnSOD, Cat, Cu/Zn SOD, HIF-α and ODH compared to PHD, GPx, MDH and HSP70, but again only reached significance in MnSOD.

Only in the German Bight population we could further compare the gene transcription response under experimentally forced and self-induced hypoxia/anoxia. Animals which self-induced anoxia for 24 h by shell closure also responded with generally decreased expression levels (Fig. 2 A–C. This response equaled the response to experimentally forced anoxia exposure. HIF-α, and MDH decreased significantly under self-induced anoxia but also Cu/Zn SOD was significantly down-regulated, which did not reach significance under experimentally forced conditions. Cat and GPx also showed decreasing expression levels under self-induced anoxia, but the effect was less clear than after 3.5 days of forced anoxia. MnSOD, PHD and HSP70 remained stable in animals with self-induced anoxia by shell closure which may relate to the short time span of 24 h. Expression changes under self-induced hypoxia/anoxia by burrowing did not merge into a common trend. Moreover, increased levels of gene transcription for Cu/Zn SOD and GPx were recorded in deep burrowing specimens compared to non-burrowing individuals (Figure S2).

### Antioxidant enzyme activities and glutathione concentration

At the protein activity level, catalase activity decreased significantly in anoxically incubated Baltic Sea and German Bight individuals. Also in German Bight animals with 24 h self-induced anoxia by shell-closure, lower catalase activities were observed (Table 4). Glutathione peroxidase activity in both populations did not change in response to different oxygen conditions (Table 4). The total glutathione content (tGSH = GSSG+GSH) increased significantly in animals from the German Bight after 24 h self-induced anoxia. Contrary, in German Bight animals that were experimentally exposed to 3.5 days hypoxia, tGSH concentrations decreased significantly, and no changes in tGSH were observed after 3.5 days of anoxia. In Baltic Sea individuals, glutathione concentrations did not change at all upon either hypoxic or anoxic incubation. Further, re-oxygenation of hypoxically and anoxically incubated Baltic Sea individuals led to a decline in tGSH in previously hypoxic animals (Table S5). Former anoxically incubated animals did not show any response in tGSH content to the re-oxygenation treatment.

**Table 4:** Activities of the antioxidant enzymes catalase and glutathione peroxidase (GPx), as well as total glutathione concentrations (tGSH = GSH+GSSG) of Baltic Sea and German Bight individuals incubated (experimentally forced) under 3.5 days of Normoxia, Hypoxia or Anoxia, or after 24 h self-induced normoxia or anoxia.

		German Bight (self-induced)			German Bight (experimentally forced)			Baltic Sea (experimentally forced)		
		Mean		S.D.	Mean		S.D.	Mean		S.D.
Catalase [U/mg protein]	Normoxia	174	±	48.81	120.4*	±	28.68	165.5*	±	16.48
	Hypoxia		±		96.82	±	22.97	**120.5**	±	73.25
	Anoxia	128.1	±	10.67	70.64*	±	29.01	84.73*	±	51.95
GPx [U/mg protein]	Normoxia	3.51	±	2.99	4.39	±	3.92	4.45	±	2.06
	Hypoxia		±		7.697	±	4.44	5.86	±	2.7
	Anoxia	3.2	±	3.13	5.21	±	3.64	6.89	±	3.34
tGSH [nmol/mg protein]	Normoxia	8.31*	±	4.12	36.86*	±	12.23	76.6	±	31.15
	Hypoxia		±		17.05*#	±	3.917	89.16	±	21.86
	Anoxia	20.57*	±	9.83	**36.87#**	±	10.19	71.39	±	33.52

Data with similar symbols (*,#) are significantly different from each other (p<0.05, one-way ANOVA and t-test). Number of n per group = 6–7 (GB tGSH Hypoxia n = 4).

### Oxidative damage and apoptosis

In Baltic Sea individuals 3.5 days of incubation under hypoxia or anoxia and subsequent re-oxygenation for 1 and 6 h did not result in accumulation of oxidized proteins. Further no induction of apoptosis could be observed under the different low oxygen and re-oxygenation treatments (Table S6).

## Discussion

Until present (2011/10/28) only 35 nucleotide and 18 protein sequences of 3 different proteins (CytB, His3, Cox1) were publicly available at Genbank and UniProt, all of which could be identified in the *A. islandica* transcriptomal data set from the present study. The newly established *A. islandica* RNAseq database consists of 35551 contigs and 98478 singletons. 66% of all assembled contigs of *A. islandica* could not be annotated by known genes from other organisms or structurally characterized by InterProScan, which highlights the scarcity of sequence information existing for marine invertebrates and *A. islandica* in particular. The resulting contigs are, however, an excellent source of putative genes especially in the context of hypoxia/anoxia-related gene regulation in *A. islandica* and will certainly be useful in future bivalve genomic research. We identified a number of contigs with high similarity to genes involved in anoxia/hypoxia tolerance, the ageing process, stress response, immune system, autophagy and apoptosis in other species. *A. islandica* is an emerging model organism for ageing research and the individual life expectancy >200 y without accumulation of major cellular damage [29], [30] calls for excellent immune system and cellular repair mechanisms. The assembled genomic information will greatly advance studies of the molecular ageing process in the ocean quahog.

### Population specific patterns of hypoxia/anoxia gene expression

Most of the hypoxia/anoxia related genes investigated in the German Bight population were down-regulated under experimentally forced anoxia and hypoxia and animals with anoxia self-induced by shell closure, whereas the opposite happened in the Baltic Sea individuals. Here, transcript levels rose in anoxia and no major changes were observed under hypoxic incubation. Gene expression and protein synthesis is energetically costly for the cells. Under oxygen limitation and MRD such processes will in most cases be suppressed for energetic reasons as reported for the snail *Littorina littorea* [31], the brine shrimp *Artemia franciscana* [32] and several hibernating mammals [33]. The general decrease in mRNA levels in hypoxia and anoxia exposed German Bight *A. islandica* is thus in agreement with this overall transcriptional suppression in animals under oxygen limiting conditions.

The investigated genes represent key genes of antioxidant defense, oxygen sensing and response initiation, as well as genes for anaerobic metabolism, all crucial for the hypoxic/anoxic survival and during re-oxygenation. We therefore suggest that in German Bight individuals hypoxia/anoxia triggers down-regulation of overall protein synthesis within the shut-down of energetic expenditures upon entering the state of metabolic rate depression (MRD). Indeed, in the land snail *Otala lactea* metabolic depression during estivation leads to a 80% reduction of protein translation rates, measured in vitro [34]. However, an overall down-regulation during metabolic depression is not always the case: In hypoxia/anoxia tolerant turtles, transcript levels of several antioxidant enzymes and heat-shock proteins are up-regulated under anoxic exposure [7]. Further, in mantle tissue of the pacific oyster *Crassostrea gigas*, the antioxidant enzyme GPx is up-regulated after 3 days of hypoxia [35]. Regarding HIF prolyl hydroxylases (PHD), in mammals these enzymes are important for oxygen sensing and control the protein levels of the hypoxia inducible transcription factor HIF-α, a master regulator of hypoxic gene transcription. PHDs and HIF-α proteins are conserved from Cnidaria to humans [36]. In mammals HIF-α regulation seems to be primarily restricted to the protein level, although some studies in humans and mice also show mRNA regulation of HIF-α under hypoxia [37], [38]. In marine invertebrates and vertebrates the investigation of protein regulation is still in its infancy due to the lack of specific antibodies for most species. For different fish species and the pacific oyster *C. gigas*, however, antibodies were generated and the mechanism of HIF regulation at the protein level and through the binding of HIF-dimer to the DNA could be observed during hypoxia [39], [40], [41]. Moreover, several studies in bivalves, shrimp and fish found that in these animals extensive HIF-α regulation takes place also on the mRNA level when animals are experiencing hypoxia or anoxia, as well as during re-oxygenation [42], [43], [44], [45]. Hypoxic exposure for 2 weeks of the eastern oyster *Crassostrea virginica* for example caused an increase in transcription levels of HIF-α and PHD in gill tissue, whereas shorter incubation (6 days) did not alter the expression levels [43], [46]. During re-oxygenation following 6 days of hypoxic exposure, however, HIF-α transcript levels increased again and significantly after 12 h re-oxygenation in *C. virginica* [43]. Also in *C. gigas* mRNA levels increased after 3 days of air exposure or 48 h incubation in hypoxic seawater [41]. Again, oyster HIF- α mRNA levels increased further upon re-oxygenation. The third group of investigated genes, the metabolic enzymes malate dehydrogenase (MDH) and octopine dehydrogenase (ODH), are crucial players in hypoxia/anoxia tolerant marine invertebrates to sustain ATP production during oxygen deficiency. Under normoxic conditions MDH plays an important role in the citrate cycle by oxidizing malate to oxalacetate whereby reducing NAD to NADH. As available oxygen decreases, marine invertebrate MDH favors the reverse reaction, reducing oxalacetate to malate by oxidizing NADH [47] and therefore replenishing the NAD pool [48]. ODHs are widely distributed among molluscs and functionally replace lactate dehydrogenases [49], [50].

The general decline in gene transcription in hypoxic/anoxic treated German Bight *A. islandica* involves all three categories (antioxidant defense, oxygen sensing, anaerobic metabolism). Thus: a down-regulation in anoxically challenged *Arctica* from the long-lived North Sea population indeed corroborates the assumption that the animals, instead of showing a stress response (HIF protein levels pending), were starting a general metabolic slow-down with reduced metabolic rate as previously shown for whole animals [6] and isolated gill tissue [14]. On the other hand, stress response enzymes such as the ones we investigated could, as described above, also be expected to be induced under some forms of stress. Larade and Storey [31] remarked that “those genes whose transcription is specifically up-regulated during anoxia stand out as genes whose protein products are likely to play very important roles in anoxia”. This represents a completely different stress response and may trigger elevation of transcript levels of the same genes in Baltic Sea *A. islandica* which might reflect an adaptation to the respective environment the animals are living in and hint towards an importance of these genes under anoxic conditions. While German Bight individuals mainly experience fully oxygenated water in their natural environment and are exposed to hypoxia and anoxia only during self-induced burrowing, Baltic Sea individuals are used to frequent environmental hypoxic and even anoxic conditions. These animals may thus show the different stress response as an adaptation to intermittent hypoxia in order to prevent oxidative stress related cellular damage. Such an adaptation has been shown in several studies investigating intertidal and subtidal animals of *Mytilus* [51], [52] or hypoxia tolerant organisms [53]. Intertidal *Mytilus* individuals showed a higher tolerance and greater response to stressors such as temperature or hypoxia compared to the subtidal individuals [51], [52]. In vertebrates, Shams et al [53] found higher, quicker and longer responses to hypoxia in hypoxia tolerant underground living mole rats (*Spalax*) compared with *Rattus*. The increase in expression levels of the investigated antioxidants, oxygen sensing and metabolic genes in Baltic Sea *A. islandica* although only marginal and not reaching significance, however, clearly demonstrate the opposite response to the German Bight population. Further, the subsequent decrease in expression levels of anoxically incubated animals within 6 h of re-oxygenation emphasizes the high flexibility in transcriptional regulation, which may be adaptive in an environment with highly fluctuation temperature, salinity and oxygen regimes. The question whether such adaptation is based on the same genotype or a mere result of phenotypic plasticity, including for example epigenetic changes, cannot be answered in the present paper. Further investigations are necessary to get a more complete picture of the hypoxic/anoxic transcription response in different *A. islandica* populations.

### Comparison of mRNA levels and protein activities related to oxidative stress

In case of catalase and glutathione peroxidase (GPx), expression changes could be compared with activity levels measured in gill homogenates. In German Bight individuals, catalase and GPx activities followed the gene expression levels with decreasing or stable values under self-induced or experimentally forced hypoxia and anoxia. In Baltic Sea individuals, the slight increase in catalase expression under anoxia was not apparent at the level of enzymatic activity. To the contrary, catalase activities decreased in anoxic compared to normoxic individuals. Thus under the current scenario of relatively short term anoxia, the increase in expression level of putative genes involved in antioxidant defense does not translate to the protein level to “prepare” the animals for the re-oxygenation [11], [54]. Indeed, the basal antioxidant capacity of the Baltic Sea *A. islandica* seems to be sufficient to prevent oxidative stress as the concentration of protein carbonyls remains stable under conditions of anoxia and re-oxygenation, and also apoptosis is not triggered. In a comparison of several North Atlantic quahog populations, Basova et al [15] found that tissue glutathione levels but not antioxidant enzyme activities were higher in BS individuals than in all other populations (including GB and Iceland). In the present study, the decrease in tGSH during re-oxygenation in former hypoxically incubated Baltic Sea individuals might be a first indication that ROS generation during re-oxygenation takes place, but can be buffered by the glutathione system to prevent cellular damage. Thus, antioxidant defense may be especially important for Baltic Sea *A. islandica* that can be exposed to prolonged periods of anoxia lasting from several days to weeks in the Baltic Sea [17], [55]. As a chronic protection from unbalanced redox conditions, glutathione levels are exaggerated compared to populations from well oxygenated environments such as the North Sea. The hypoxic/anoxic exposure in our experiments may not have been long enough to induce stress gene transcription, which is costly. Next to the ATP requirement for providing the nucleotide substrates, also the transcriptional machinery needs energy resources [31]. These expenses draw on the energy reserves of the Baltic Sea individuals during anoxia, which may reduce the energy saving effect of self induced metabolic slow down during MRD [56], [57] and reduce life span compared to the German Bight and, presumably, the Iceland populations. Further experiments with longer incubation times need to reveal whether the initiation of enhanced transcript levels in Baltic Sea individuals will be sustained under prolonged anoxia and eventually translate into higher antioxidant activities.

Oxygen concentration is however only one environmental factor which might determine the different MLSPs. The shorter lived populations (Baltic Sea, White Sea, Kattegat) are further exposed to fluctuating temperature and salinity regimes [15]. Basova et al. [15] investigated antioxidant capacity and basal metabolic rates in 5 different *A. islandica* populations with different MLSPs and related the results to the ecological conditions in each respective environment of origin. Shorter lived populations of the Baltic Sea, White Sea and Kattegat displayed higher basal metabolic rates compared to longer lived populations of the North Sea and Iceland, while antioxidant enzyme capacities were similar between the 5 populations [15]. The authors proposed that the higher metabolic rates imply a more flexible thermal response, needed in highly fluctuating environments. MLSP in the 5 populations was also negatively correlated with the annual salinity amplitude in the different marine environments. We thus hypothesize that *A. islandica* populations from more fluctuant marine/brackish environments of the Baltic and White Seas may need to allocate higher energy resources to stress response and regulation compared to animals of populations living in more stable environments. In respect to the theory of hormesis, variable environments can also be beneficial for longevity [58], [59], which contrasts our observations. However the concept of hormesis refers to mild stress, mostly at young age, which can lead to increased longevity. The definition of mild stress needs, however, to be evaluated for a specific species in a specific environmental setting. In Baltic Sea *A. islandica* we hypothesize that the environmental stress is too severe to have an beneficial effect on longevity.

Further, regional and seasonal cold climates favour states of metabolic rate depression in bivalves like the Russian pearl clam *Margaritifera margaritifera* [60] or the Antarctic soft shell clam *Laternula elliptica* [61]. At the same time this increases survival under low oxygen conditions [62] and increases the effect of anoxic exposure on metabolic enzymes properties [63], [64]. Indeed, Baltic Sea *A. islandica* kept for 1 year at higher salinity (25–35psu) and low temperatures (4–10°C) showed higher growth rates and better condition index, featured lower mortality and less accumulation of cellular damage (lipofuscin) compared to individuals kept at low salinity (15 psu) and high temperature (16°C) [65].

## Conclusions

Several contigs with putative function important for hypoxia/anoxia response, general stress response as well as related to the ageing process, immune system and cellular homeostasis were identified in the newly generated *A. islandica* RNAseq database. The investigation of expression changes of putative antioxidant, oxygen sensing and metabolic genes under different oxygen conditions revealed transcription suppression in all three gene groups in anoxic incubated individuals of the German Bight population and increased transcription in the Baltic Sea population. The results indicate population specific transcriptional responses to low oxygen, which might relate to the different environments the populations are living in. Individuals from the Baltic Sea live in a highly fluctuating environment with fast and extreme changes in temperature, salinity and oxygen, whereas German Bight individuals experience a more stable environment. Adaptation to Baltic Sea conditions might have led to higher physiological flexibility and stress hardening in this *A. islandica* population. Higher fluctuations involving limiting but still subcritical conditions such as prolonged anoxia in the quahog may cause the animals to advance onset of reproduction (and perhaps also intensify reproductive efforts) and to invest more into fitness and stress defense capacities in young animals at the cost of maximum lifespan. The overall conclusion from these investigations in the long-lived ocean quahog seems to be therefore that lifespan in this animal is not only a genetic feature, but also depends on phenotypic adaptation to the levels of environmental variability in a specific habitat.

## Supporting Information

Figure S1 **Read length (A) and contig length (B) distribution in the** ***Arctica islandica*** **transcriptome database.**
(DOC)

Figure S2 **Quantitative expression of candidate genes for German Bight** ***A. islandica*** **individuals with self-induced normoxia (non-burrowing) and hypoxia/anoxia by burrowing for 3.5 days (burrowing).** Oxygen concentration of the burrowing animals was not measured and oxygen conditions can only be assumed due to burrowing activity and accumulation of anaerobic end products as described for the same individuals in Strahl et al [5]. Expression was assessed by q-RT PCR and levels normalized using the geometric mean of 18S, 28S, HSP90, which were selected as most stable reference genes by Normfinder. N = 7. Values are significantly different with *p<0.05, ** p<0.01 (t-test).
(TIF)

Table S1 **Details of number and length of sequences generated by 454 technology and used for the transcriptome generation before and after cleaning.**
(DOC)

Table S2 **Forward and reverse primer sequences used for semi-quantitative PCR and Sanger sequencing.**
(DOC)

Table S3 **General characteristics of candidate genes important for anoxia/hypoxia tolerance and general stress response identified in the** ***A. islandica*** **transcriptome.** Given are the expected domains of the respective gene (deduced from homologene http://www.ncbi.nlm.nih.gov/sites/entrez/query.fcgi?db=homologene) and observed domains in the contig (by SMART http://smart.embl-heidelberg.de/ and transmembrane domains re-checked by THMM http://www.cbs.dtu.dk/services/TMHMM-2.0/), as well as length of the corresponding open-reading frame (ORF).
(DOC)

Table S4 **Contigs of** ***A. islandica*** **which show high similarity to candidate genes involved in the ageing process and immune system functioning.** Given are the expected domains of the respective gene (deduced from homologene http://www.ncbi.nlm.nih.gov/sites/entrez/query.fcgi?db=homologene) and observed domains in the contig (by SMART http://smart.embl-heidelberg.de/ and transmembrane domains re-checked by THMM http://www.cbs.dtu.dk/services/TMHMM-2.0/), as well as contig length, the read number the contig was assembled from and the longest open-reading frame (ORF). Further the best BLAST hit from the UniprotKB/SwissProt and the NCBI nt database is given.
(XLS)

Table S5 **Activities of the antioxidant enzymes catalase and glutathione peroxidase (GPx), as well as total glutathione concentrations (tGSH = GSH+GSSG) in gill tissue of Baltic Sea** ***A. islandica*** **individuals exposed for 3.5 days to Normoxia, Hypoxia and Anoxia and after 1 and 6 hours re-oxygenation.** Data with similar symbols (*,#) are significantly different from each other (p<0.05, one-way ANOVA). Number of n per group = 6–8.
(DOC)

Table S6 **Concentration of protein carbonyls [nmol/mg] and caspase activity [RLU/mg protein] in gill tissue of Baltic Sea** ***A. islandica*** **individuals exposed for 3.5 days to Normoxia, Hypoxia and Anoxia and after 1 and 6 hours re-oxygenation.** Number of n per group = 6–8.
(DOC)
