# Supplementary figures and images for: Gene Expression and Physiological Changes of Different Populations of the Long-Lived Bivalve Arctica islandica under Low Oxygen Conditions
Source: PLoS One. 2012 Sep 19;7(9):e44621. doi: 10.1371/journal.pone.0044621 (PMC3446923; doi:10.1371/journal.pone.0044621)

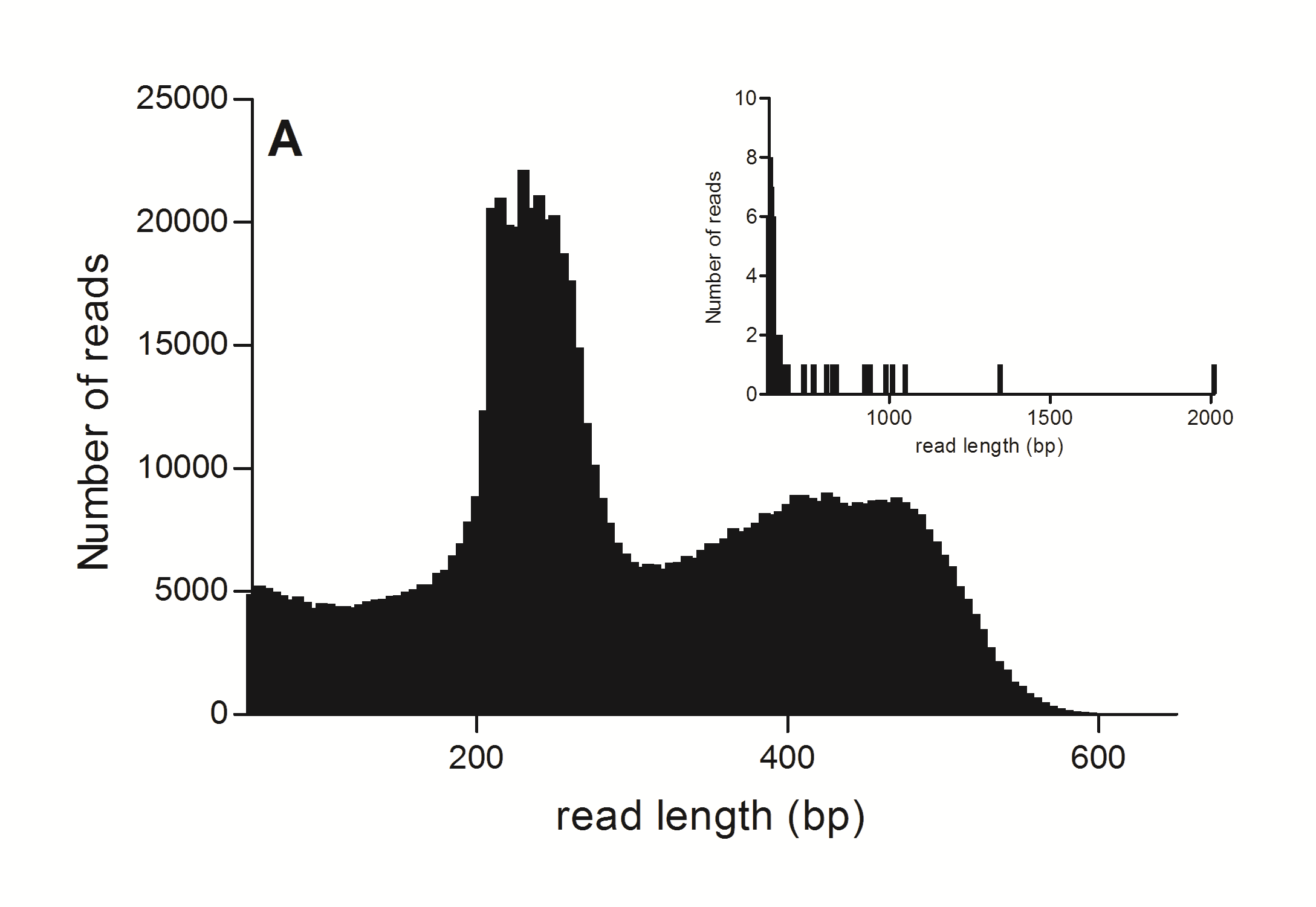


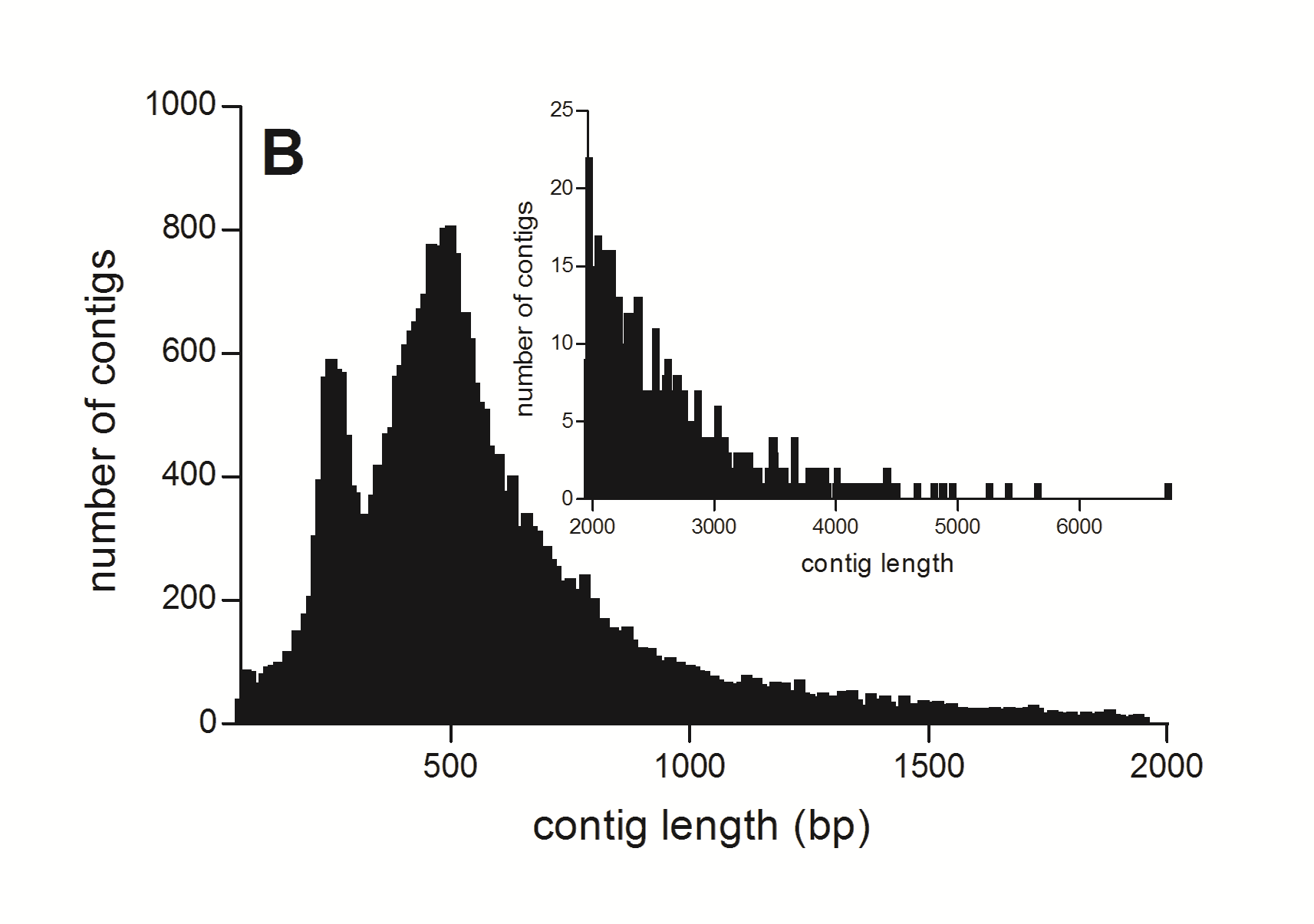

Supplement: Figure S1 — Read length (A) and contig length (B) distribution in the Arctica islandica transcriptome database. (DOC) [file pone.0044621.s001.doc]

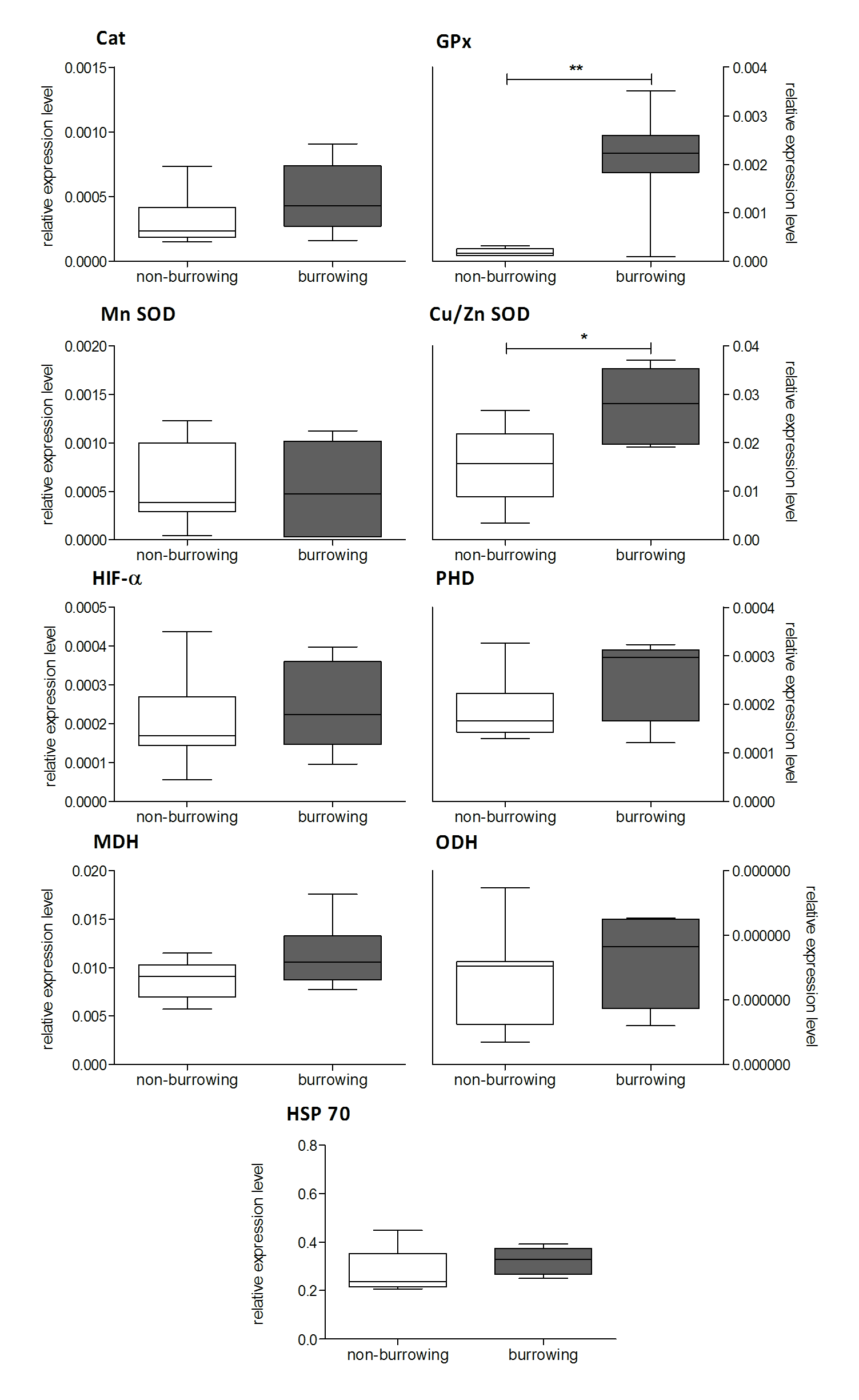

Supplement: Figure S2 — Quantitative expression of candidate genes for German Bight A. islandica individuals with self-induced normoxia (non-burrowing) and hypoxia/anoxia by burrowing for 3.5 days (burrowing). Oxygen concentration of the burrowing animals was not measured and oxygen conditions can only be assumed due to burrowing activity and accumulation of anaerobic end products as described for the same individuals in Strahl et al [5]. Expression was assessed by q-RT PCR and levels normalized using the geometric mean of 18S, 28S, HSP90, which were selected as most stable reference genes by Normfinder. N = 7. Values are significantly different with *p<0.05, ** p<0.01 (t-test). (TIF) [file pone.0044621.s002.tif]
