# Supplementary material for: Gene Expression and Physiological Changes of Different Populations of the Long-Lived Bivalve Arctica islandica under Low Oxygen Conditions
Source: PLoS One. 2012 Sep 19;7(9):e44621. doi: 10.1371/journal.pone.0044621 (PMC3446923; doi:10.1371/journal.pone.0044621)
Supplement: Table S1 — Details of number and length of sequences generated by 454 technology and used for the transcriptome generation before and after cleaning. (DOC) [file pone.0044621.s003.doc]

Table S1:

|  | Roche sff file before quality clip and cleaning | | | | | | After quality clip and cleaning | | | | | |
| --- | --- | --- | --- | --- | --- | --- | --- | --- | --- | --- | --- | --- |
| Filename | Nr Sequences | Average Length (bp) | Max. Length (bp) | Min. Length (bp) | Mb | N50 | Nr Sequences | Average Length (bp) | Max. Length (bp) | Min. Length (bp) | Mb | N50 |
| 144_A_islandica_gill_control_080724.fasta | 45213 | 218 | 382 | 21 | 9,87 | 249 | 41491 | 210 | 350 | 51 | 8,72 | 238 |
| 145_A_islandica_DG_stress_080724.fasta | 5703 | 225 | 383 | 20 | 1,28 | 246 | 5364 | 209 | 325 | 51 | 1,12 | 222 |
| 146_A_islandica_gill_stress_080724.fasta | 59053 | 241 | 434 | 29 | 14,27 | 257 | 57851 | 231 | 355 | 51 | 13,41 | 246 |
| 147_A_islandica_DG_control_080724.fasta | 31378 | 236 | 481 | 10 | 7,42 | 249 | 30673 | 223 | 354 | 51 | 6,84 | 235 |
| 160_A_islandica_081124_Titration05.fasta | 675 | 217 | 324 | 32 | 0,14 | 246 | 630 | 206 | 294 | 54 | 0,13 | 227 |
| 160_A_islandica_081124_Titration06.fasta | 1216 | 218 | 401 | 32 | 0,26 | 249 | 1123 | 209 | 351 | 52 | 0,23 | 227 |
| 160_A_islandica_081124_Titration07.fasta | 1535 | 223 | 320 | 19 | 0,34 | 250 | 1412 | 214 | 313 | 52 | 0,3 | 227 |
| 160_A_islandica_081124_Titration08.fasta | 3509 | 202 | 428 | 30 | 0,7 | 244 | 3140 | 198 | 329 | 51 | 0,62 | 224 |
| 160_A_islandica_090109_Titration16.fasta | 7451 | 193 | 331 | 25 | 1,44 | 238 | 6623 | 191 | 312 | 51 | 1,26 | 218 |
| 160_A_islandica_fullProcessing _Reanalysis_090122.fasta | 158075 | 224 | 415 | 17 | 35,49 | 248 | 147336 | 212 | 346 | 51 | 31,26 | 227 |
| 196_Arctica_islandica_old_20091012.fasta | 277252 | 321 | 1077 | 40 | 89,07 | 420 | 249099 | 327 | 1047 | 51 | 81,47 | 403 |
| 197_Arctica_islandica_young_20091012.fasta | 281206 | 357 | 2044 | 40 | 100,63 | 434 | 264585 | 354 | 2006 | 51 | 93,85 | 415 |
| *Mean or *Total* | *872266** | *240* | *585* | *26* | *261** | *278* | *809327** | *232* | *532* | *51* | *239** | *259* |
