# Supplementary material for: Gene Expression and Physiological Changes of Different Populations of the Long-Lived Bivalve Arctica islandica under Low Oxygen Conditions
Source: PLoS One. 2012 Sep 19;7(9):e44621. doi: 10.1371/journal.pone.0044621 (PMC3446923; doi:10.1371/journal.pone.0044621)
Supplement: Table S2 — Forward and reverse primer sequences used for semi-quantitative PCR and Sanger sequencing. (DOC) [file pone.0044621.s004.doc]

Table S2:

| *Cantidate gene* | *ID/ Accession* | *Contig length (bp)* | 5´-3´ forward primer | 5´-3´ reverse primer | Amplicon length (bp) |
| --- | --- | --- | --- | --- | --- |
| Catalase | HE792873 | 2145 | TGATTTATATCAAATACACATTCTTCG | AGTTGTTATCTAATTCAACAAAACTCG | 2132 |
| Glutathione peroxidase (GPX) | HE792874 | 1210 | GTTTCGGGGCTCATCATTT | GCATTCTTTTATTGTTTATTTCAGTCC | 1032 |
| Superoxide dismutase (Cu/Zn SOD) | HE792875 | 1934 | GTCTTTAAATGTCTCAATTGCACAAC | TTTAAAGTAAACAAAACAACAGTCAGA | 1846 |
| Superoxide dismutase (Mn SOD) | HE792877 | 1682 | TACCACTAAAATTTGTAAATTCCCATT | GAGGACAGTACTGAACAGCCTTG | 1649 |
| Octopine dehydrogenase (ODH) | HE792878 | 461 | TTCGGGGGTCACTCTCTC | AACTACGCAAGAGTCCAGCAA | 461 |
| Malate dehydrogenase (MDH) | HE792879 | 1497 | TTGAGTATAGCTACAGCACTTTTATCA | GGGGGTTCCTTAAGGTCAA | 1458 |
| Hypoxia inducible factor alpha (HIF alpha) | HE792881 | 4322 | TTATTCGTTTATTGTTTATTTCAGTCC | GACTTGGGAAATATTTTGGTTTG | 4278 |
| HIF prolyl hydroxylase (PHD) | HE792884 | 567 | CGACTTAGCGATTTCCAGGT | CCTTCAGAGACGGGACAGC | 402 |
| HSP70 | HE792888 | 2379 | CCCCAGGCTCAGTTAGTTTT | TTTCGGGGGAGTTGTTACT | 2379 |
| HSP90 | HE792890 | 2826 | AGATGAAAATGAATGGCAGAAA | CAGGCGGAGATTGCTCAGT | 2566 |
