# Supplementary material for: Gene Expression and Physiological Changes of Different Populations of the Long-Lived Bivalve Arctica islandica under Low Oxygen Conditions
Source: PLoS One. 2012 Sep 19;7(9):e44621. doi: 10.1371/journal.pone.0044621 (PMC3446923; doi:10.1371/journal.pone.0044621)
Supplement: Table S3 — General characteristics of candidate genes important for anoxia/hypoxia tolerance and general stress response identified in the A. islandica transcriptome. Given are the expected domains of the respective gene (deduced from homologene http://www.ncbi.nlm.nih.gov/sites/entrez/query.fcgi?db=homologene) and observed domains in the contig (by SMART http://smart.embl-heidelberg.de/ and transmembrane domains re-checked by THMM http://www.cbs.dtu.dk/services/TMHMM-2.0/), as well as length of the corresponding open-reading frame (ORF). (DOC) [file pone.0044621.s005.doc]

Table S3:

| *Category* | *Gene name* | *Function* | *Accession* | *ORF (aa)* | *Domains expected* | *Domains observed* |
| --- | --- | --- | --- | --- | --- | --- |
| Hypoxia/Anoxia | Catalase | *antioxidant enzyme* | HE792873* | 513 | catalase domain | catalase |
|  | Glutathione peroxidase (GPX) | *antioxidant enzyme* | HE792874* | 166 | thioredoxin like domain | GSHPx |
|  | Superoxide dismutase (Cu/Zn SOD) | *antioxidant enzyme* | HE792875* | 285 | Cu-ZN_superoxide dismutase | Sod_Cu, TM |
|  |  | *antioxidant enzyme* | HE792876 | 154 | Cu-ZN_superoxide dismutase | Sod_Cu |
|  | Superoxide dismutase (Mn SOD) | *antioxidant enzyme* | HE792877* | 226 | Sod_Fe_N, Sod_Fe_C | Sod_Fe_N, Sod_Fe_C |
|  | Octopine dehydrogenase (ODH) | *metabolic enzyme* | HE792878* | 109 | NAD_Gly3P_dh_N, Octopine_DH | - |
|  | Malate dehydrogenase (MDH) | *metabolic enzyme* | HE792879* | 331 | LDH_MDH_like | Ldh_1_N, Ldh_1_C |
|  |  | *metabolic enzyme* | HE792880 | 363 | LDH_MDH_like | Ldh_1_N, Ldh_1_C |
|  | Hypoxia inducible factor alpha (HIF alpha) | *hypoxia sensing* | HE792881* | 771 | HLH, PAS, Pas_3, HIF_1a_CTAD, HIF-1 | HLH, PAS, PAS, PAC |
|  | Aryl hydrocarbon receptor nuclear translocator (HIF beta / ARNT) | *hypoxia sensing* | HE792882 | 239 | HLH, PAS, Pas_3, | PAS, PAC |
|  | Von Hippel-Lindau (VHL) | *hypoxia sensing* | HE792883 | 114 | pVHL | VHL |
|  | HIF prolyl hydroxylase (PHD) | *hypoxia sensing* | HE792884* | 108 | zf-MYND, 2OG-FeII_Oxy | 2OG-FeII_Oxy |
|  | Peroxiredoxin | *antioxidant enzyme* | HE792885 | 193 | Thioredoxin-like | AhpC-TSA/Redoxin |
|  |  | *antioxidant enzyme* | HE792886 | 222 | Thioredoxin-like | AhpC-TSA/Redoxin, 1-cysPrx_C |
|  | Thioredoxin | *antioxidant enzyme* | HE792887 | 144 | Thioredoxin-like | AhpC-TSA/Thioredoxin |
| Stress response | HSP70 ^a^ | *chaperone* | HE792888* | 655 | Heat shock 70 kDa protein | Hsp70 |
|  |  | *chaperone* | HE792889 | 570 | Heat shock 70 kDa protein | Hsp70 |
|  | HSP90 ^a^ | *chaperone* | HE792890* | 735 | Heat shock protein 90, Histidine kinase-like ATPase (HATPase_c) | HATPase_c, HSP90 |
|  |  | *chaperone* | HE792891 | 276 | Heat shock protein 90, Histidine kinase-like ATPase (HATPase_c) | HSP90 |
|  |  | *chaperone* | HE792892 | 304 | Heat shock protein 90, Histidine kinase-like ATPase (HATPase_c) | HATPase_c, 2x HSP90 |

^a^For HSPs only contigs with >100reads were selected for display. Additional contigs with high similarity to HSPs are present in the transcriptome.

*Genes investigated for differential expression in *A. islandica* gill tissue of animals incubated under different oxygen regime
