# Supplementary material for: Gene Expression and Physiological Changes of Different Populations of the Long-Lived Bivalve Arctica islandica under Low Oxygen Conditions
Source: PLoS One. 2012 Sep 19;7(9):e44621. doi: 10.1371/journal.pone.0044621 (PMC3446923; doi:10.1371/journal.pone.0044621)
Supplement: Table S5 — Activities of the antioxidant enzymes catalase and glutathione peroxidase (GPx), as well as total glutathione concentrations (tGSH = GSH+GSSG) in gill tissue of Baltic Sea A. islandica individuals exposed for 3.5 days to Normoxia, Hypoxia and Anoxia and after 1 and 6 hours re-oxygenation. Data with similar symbols (*,#) are significantly different from each other (p<0.05, one-way ANOVA). Number of n per group = 6–8. (DOC) [file pone.0044621.s007.doc]

Table S5:

|  | Catalase [U/mg protein] | | | GPx [U/mg protein] | | | tGSH [nmol /mg protein] | | |
| --- | --- | --- | --- | --- | --- | --- | --- | --- | --- |
|  | Mean |  | S.D. | Mean |  | S.D. | Mean |  | S.D. |
| Normoxia | **165.5^*#^** | ± | 16.48 | 4.45 | ± | 2.06 | 76.60 | ± | 31.15 |
| Normox-1h control | **83.28^#^** | ± | 55.23 | 6.70 | ± | 3.49 | 62.80 | ± | 22.12 |
| Normox 6h control | 117.70 | ± | 50.27 | 6.74 | ± | 3.38 | 73.13 | ± | 13.84 |
| Hypoxia | 120.50 | ± | 73.25 | 5.86 | ± | 2.69 | **89.16^*^** | ± | 21.86 |
| Hypox-reox 1h | 98.26 | ± | 71.40 | 6.15 | ± | 2.87 | 66.99 | ± | 16.00 |
| Hypox-reox 6h | 105.30 | ± | 58.71 | 8.41 | ± | 3.36 | **57.24^*^** | ± | 20.96 |
| Anoxia | **84.73^*^** | ± | 51.95 | 6.89 | ± | 3.33 | 71.39 | ± | 33.52 |
| Anox - reox 1h | 108.50 | ± | 52.07 | 7.14 | ± | 4.05 | 67.84 | ± | 13.79 |
| Anox - reox 6h | 129.00 | ± | 49.22 | 6.74 | ± | 3.17 | 68.29 | ± | 30.37 |
