# Supplementary material for: Gene Expression and Physiological Changes of Different Populations of the Long-Lived Bivalve Arctica islandica under Low Oxygen Conditions
Source: PLoS One. 2012 Sep 19;7(9):e44621. doi: 10.1371/journal.pone.0044621 (PMC3446923; doi:10.1371/journal.pone.0044621)
Supplement: Table S6 — Concentration of protein carbonyls [nmol/mg] and caspase activity [RLU/mg protein] in gill tissue of Baltic Sea A. islandica individuals exposed for 3.5 days to Normoxia, Hypoxia and Anoxia and after 1 and 6 hours re-oxygenation. Number of n per group = 6–8. (DOC) [file pone.0044621.s008.doc]

Table S6

|  | Protein carbonyls [nmol/mg] | | | Caspase activity [RLU/mg protein] | | |
| --- | --- | --- | --- | --- | --- | --- |
|  | Mean |  | S.D. | Mean |  | S.D. |
| Normoxia | 2.603 | ± | 0.8514 | 60421 | ± | 18177 |
| Normox-1h control | 2.075 | ± | 0.3869 | 50832 | ± | 16068 |
| Normox 6h control | 2.918 | ± | 0.922 | 64182 | ± | 25534 |
| Hypoxia | 3.701 | ± | 1.279 | 52774 | ± | 35446 |
| Hypox-reox 1h | 2.822 | ± | 1.219 | 55856 | ± | 19915 |
| Hypox-reox 6h | 3.564 | ± | 0.9994 | 53787 | ± | 12499 |
| Anoxia | 2.373 | ± | 0.5799 | 46094 | ± | 13160 |
| Anox - reox 1h | 2.978 | ± | 1.283 | 42980 | ± | 12614 |
| Anox - reox 6h | 2.718 | ± | 1.535 | 61694 | ± | 22231 |
